# MSCs and Inflammatory Cells Crosstalk in Regenerative Medicine: Concerted Actions for Optimized Resolution Driven by Energy Metabolism

**DOI:** 10.3389/fimmu.2021.626755

**Published:** 2021-04-30

**Authors:** Valerie Planat-Benard, Audrey Varin, Louis Casteilla

**Affiliations:** RESTORE, University of Toulouse, UMR 1031-INSERM, 5070-CNRS, Etablissement Français du Sang-Occitanie (EFS), Université Paul Sabatier, Toulouse, France

**Keywords:** mesenchymal stromal cells (MSCs), inflammation, macrophage, immunomodulation, metabolic reprogramming, cell therapy, regenerative medicine

## Abstract

Mesenchymal stromal cells (MSCs) are currently widely used in cell based therapy regarding to their remarkable efficacy in controlling the inflammatory status in patients. Despite recent progress and encouraging results, inconstant therapeutic benefits are reported suggesting that significant breakthroughs in the understanding of MSCs immunomodulatory mechanisms of action remains to be investigated and certainly apprehended from original point of view. This review will focus on the recent findings regarding MSCs close relationship with the innate immune compartment, i.e. granulocytes and myeloid cells. The review will also consider the intercellular mechanism of communication involved, such as factor secretion, cell-cell contact, extracellular vesicles, mitochondria transfer and efferocytosis. Immune-like-properties of MSCs supporting part of their therapeutic effect in the clinical setting will be discussed, as well as their potentials (immunomodulatory, anti-bacterial, anti-inflammatory, anti-oxidant defenses and metabolic adaptation…) and effects mediated, such as cell polarization, differentiation, death and survival on various immune and tissue cell targets determinant in triggering tissue regeneration. Their metabolic properties in term of sensing, reacting and producing metabolites influencing tissue inflammation will be highlighted. The review will finally open to discussion how ongoing scientific advances on MSCs could be efficiently translated to clinic in chronic and age-related inflammatory diseases and the current limits and gaps that remain to be overcome to achieving tissue regeneration and rejuvenation.

## Introduction

Mesenchymal stromal cells (MSCs) are present in all tissues as a major structural and connective component sensing microenvironment signals and acting to maintain tissue homeostasis. They represent a heterogeneous cell population among which the presence of a canonic mesenchymal stem cell subpopulation is largely questioned. The role of such a multipotent progenitor subpopulation is out of the scope of the present review that will be focused on the stromal and supportive functions of MSCs. Cell-based therapy harnesses these activities to orchestrate tissue reconstruction and rescue functional and structural integrity upon injury and disease. Recent findings outline that metabolism is driving MSCs mobilization and responses and that immune cells, also present in all tissues, remain one of the privileged targets, acting together for driving the therapeutic outcome. Based on clinical results using MSCs as advanced medicinal products (ATMP) a focus will be made on the immunomodulatory effect observed in various regenerative medicine applications. The object of the present review is thus to provide an overview of the recent progress in deciphering cellular, molecular and metabolic communication between MSCs and innate immunity cells. The positioning of MSCs as a component of the immune system will be questioned. We will discuss how MSCs and macrophages organize into a highly interconnected network where cell metabolism and energy management reflect and modulate both mesenchymal and immune functions. Finally, the discussion will open to novel consideration of such metabolic immune-mesenchymal axis to maintain or restore tissue homeostasis.

## Clinical Outcomes from the use of MSCs in Inflammatory/Immune Diseases

MSCs are good candidates for cell therapy to treat inflammatory diseases such as graft versus host disease (GVHD) (1), sepsis (2) and inflammatory bowel diseases (IBD) (3, 4) based on their capability to resolve inflammation and promote tissue repair by modulating endogenous tissue and immune cells such as macrophages, neutrophils, and natural killer (NK) cells.

The first therapeutic use of MSCs was to treat severe GVHD. Severe GVHD is a life-threatening complication after allogeneic transplantation with hematopoietic stem cells (HSCs) that is characterized by the activation of the immune system with systemic inflammation and tissue injury. Steroids are the first-line treatment for established GVHD however, 50% of patients are non-responders and have a poor outcome (5). MSCs support proliferation and differentiation of HSCs in the bone marrow, promote HSCs engraftment in animal model and can decrease inflammation (6). Therefore, MSCs were seen as good candidates to treat steroid-resistant acute GVHD (SR-aGVHD). In a phase II multicenter European study, 55 patients with steroid-resistant acute severe GVHD were treated with bone marrow-derived MSCs (BM-MSCs). 39 out of the 55 patients responded to the treatment and the survival of patients with complete response were significantly higher and transplantation-related mortality after injection was significantly lower than in non- or partial responder patients (7). Moreover, in a phase III, prospective, single-arm, multicenter study, pediatric patients with primary SR-aGVHD where treated with allogenic BM-MSCs. This study demonstrated that MSCs infusions significantly improve overall response in pediatric patients with SR-aGVHD compared with derived historical rates and are well tolerated with no safety issues. In addition, the observed improved response at day 28 was strongly associated with significantly improved survival through day 180 (8). This report supports previous studies that demonstrated the beneficial effect of BM-MSCs on SR-GVHD (9, 10). However, a recent meta-analysis questioned the positive effect of MSCs on GVHD. Indeed, injection of MSCs can decrease chronic GVHD but not protect from acute GVHD. Similarly, another meta-analysis demonstrated that the studies analyzing the effect of MSCs on GVHD are low-quality evidence and the results of the current published random clinical trials do not support the conclusion that MSCs are effective therapy for GVHD (11). Such divergences may come from the time of transplantation. Indeed, umbilical-derived MSCs (UC-MSCs) and MSCs infused after HSCs can reduce chronic GVHD, favor HSCs engraftment and decrease incidence rate of relapse and death whereas treatment with BM-MSCs and MSCs before HSCs transplantation have a negative effect on patient survival (12). Taken together, the absence of robust effect of MSCs, due to the small number of studies and the limited number of enrolled patients indicate that high-quality controlled clinical trial as well as a more refined understanding of MSCs action are required to prove the beneficial effect of MSCs on GVHD treatment.

More robust results were obtained in inflammatory bowel diseases (IBD), Crohn’s disease and ulcerative colitis where MSCs demonstrate a great efficacy. IBD is characterized by a chronic and destructive inflammation of the gastrointestinal tract. Indeed, IBD pathogenesis is associated with IL-23 and IL-12 pro-inflammatory cytokines that promote in the intestine the switch of T lymphocytes toward a Th1 pro-inflammatory profile and the formation of Th17 lymphocytes playing an important role in inflammation and tissue damage in autoimmune diseases such as Crohn diseases (13). The Th17 T cells produce IL-17 that locally stimulates chemokine production attracting macrophages, neutrophils and increasing local inflammation (14). In a different experimental model, administration of MSCs protects against colitis by regulating the Th1/Th17 response, increasing the number of FoxP3 positive regulatory T cells in the colon and in the mesenteric lymph node. MSCs also modulate inflammation by decreasing pro-inflammatory cytokines such as TNF-α, IL-12 and IFN-γ and increasing IL-10 in the colon (15, 16). MSCs seem to target directly the Th-17 T cells increasing their expression of FoxP3 mRNA, switching them in regulatory T cells, inhibiting their production of inflammatory cytokines. In 2018, the European Commission approved the first MSCs pharmaceutical product to treat Crohn’s-related enterocutaneous fistular diseases, following several clinical trials demonstrating that local injection of BM-MSCs (17) or ASCs (18, 19) can induce remission in patients that previously failed with classical therapies. More than 50% of the MSCs-treated patients present a complete closure of all fistula tracts that was sustained at 52 weeks post-treatment. In addition, beneficial effects of MSCs were demonstrated in ulcerative colitis (20).

Despite the absence of phase 3 clinical trial, *in vivo* preclinical and phase 1 clinical studies seem to demonstrate that MSCs could also be a new treatment for acute inflammatory diseases such as sepsis (21). Sepsis is a life-threatening syndrome resulting in shock and multiple organ dysfunction due to microbial infection. The loss of immune homeostasis induced by the presence of the pathogens drive a hyperproduction of pro-inflammatory cytokines as well as anti-inflammatory molecules by the innate immune cells that induce the “cytokine storm” that is associated with the multi-organ failure and death. A meta-analysis demonstrated that in preclinical sepsis models, treatment with MSCs decreases drastically the mortality and the general inflammation of septic animals (22). In human, several phase I clinical trials concluded that intravenous injection of umbilical, bone marrow- or adipose tissue-derived MSCs is safe and does not induce harmful side effect on patients (23–25). In a single-center and randomized study, one intravenous injection of MSCs in severe neutropenic patients with septic shock reduced the Sequential Organ Failure Assessment score and significantly increased survival rates compared to the conventional treatment group. However, MSCs treatment did not prevent long-term death from sepsis-related organ failures (26). Moreover, several studies demonstrated that intravenous injection of MSCs can modify the immune response during sepsis. Indeed, intravenous injection of allogenic BM-MSCs or adipose-derived mesenchymal stromal cells (ASCs) on septic shock patients had none or mild effect on increased pro-inflammatory cytokines and biomarkers but MSCs injection induced a time-depend decreased of pro-inflammatory cytokines as well as pro-coagulant factors in patients on septic shock (24, 27).

Similarly, multiple human clinical studies reported that MSCs administration is safe for patients with severe pulmonary diseases such as acute respiratory distress syndrome (ARSD), acute lung injury (ALI) (25, 28) or chronic obstructive pulmonary disease (COPD). However, due to the relatively small number of patients enrolled in clinical trial, efficacy of MSCs treatment for severe pulmonary diseases has not been demonstrated yet (29). Interestingly, a recent study demonstrated safety and efficacy of MSCs as treatment of Influenza A (H7N9)-induced ARDS (30). Indeed, the MSCs-treated group have a significantly higher survival rate compared to the control group. At the 1 year follow-up, MSCs-treated patients have an improvement of chest CT scans and they presented no decline in pulmonary functions nor deleterious effects link to the treatment. Therefore, MSCs have been rationally considered and already tested as a possible treatment for Covid-19 (31). Despite great progress in the knowledge of MSCs biology, conclusions from clinical trials using MSCs in inflammatory diseases reveal that no adverse events are reported and beneficial effect can be expected even though consistent and robust clinical improvement is not yet reached. Not all patients respond to treatment suggesting that there is more to understand about their mechanisms of action. To that end their broad reactivity with the immune system remain to be explored to provide substantial advance in MSCs therapy.

## MSCs and Inflammatory Response: a Question oF cell Dialogue

The immunomodulatory potential of MSCs is thus supported by experimental and clinical data and rely on an organized and complex network interfacing immune cells with MSCs. While immunosuppressive activity of MSCs on adaptive immune cells is already largely documented (32), and in line with conclusions from clinical trial findings, we will focus here on innate immune system cells.

**Figure 1:**
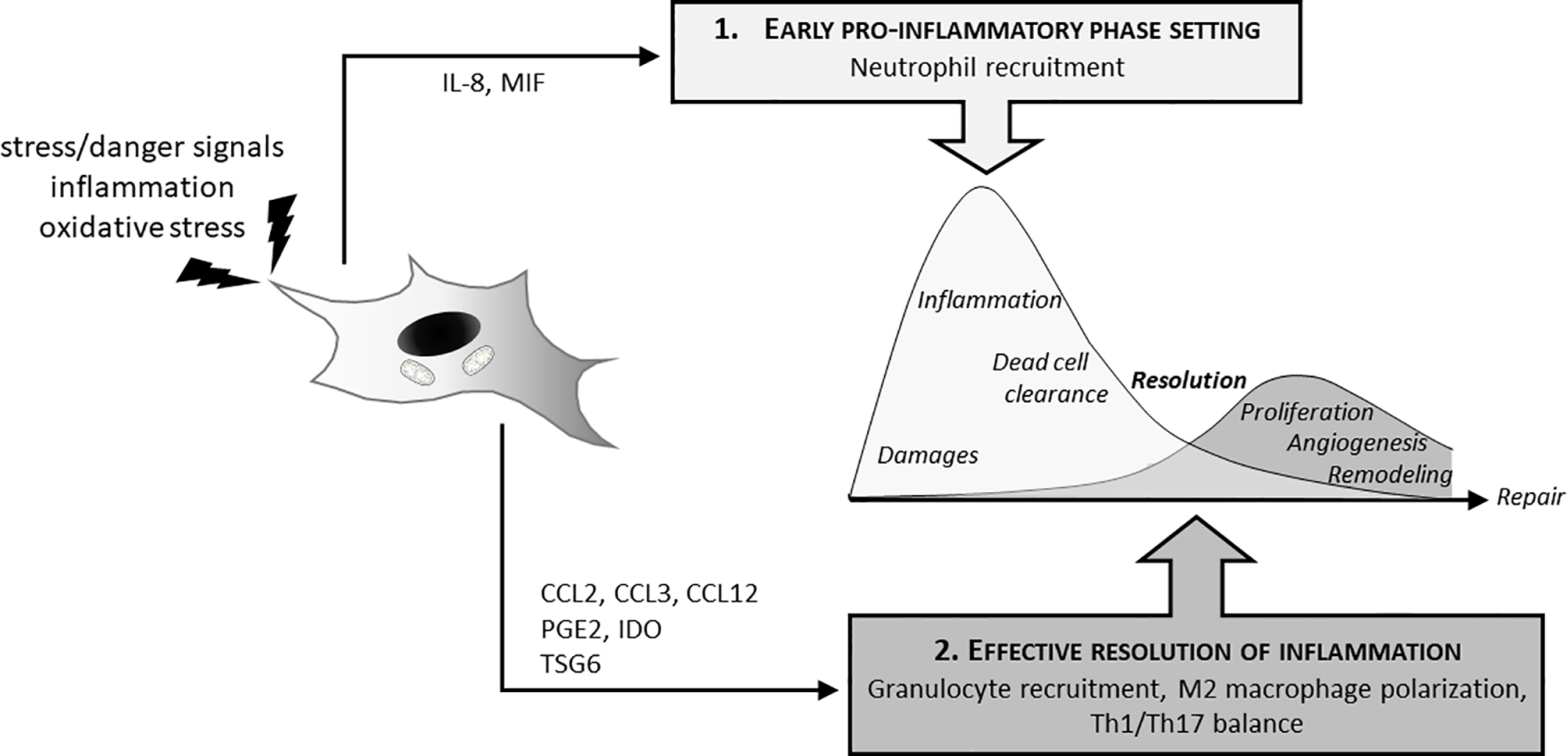
MSCs and inflammatory response: dual effect on induction and resolution of the inflammation. In the initial phase of the inflammatory process, MSCs actively participate to neutrophil attraction (IL8, MIF secretion) for anti-bacterial effect and clearance of tissue debris. This initial phase is required to further promote tissue recovery and repair. MSCs also have decisive effects on the switch from this inflammatory phase to its resolution necessary to initiate tissue reconstruction. MSCs thus favor granulocyte recruitments through cytokines (CCL2, CCL3, CCL12), induce M2 polarization (PGE2, IDO, TSG6) as well as Th1/Th17 balance.

MSCs play a critical role in the two phases of inflammation essential for an effective healing process: the early acute phase that initiate inflammation and its subsequent resolution necessary to achieve the repair process (**Figure 1**). Neutrophils are the first line of defense against pathogens and tissue injury that are massively recruited to the site of infection or damaged tissue. Several papers functional interaction between MSCs and neutrophils allows an efficient mobilization of immune cells. LPS (lipopolysaccharide)-stimulated glandular MSCs increased the recruitment of polymorphonuclear leucocytes through the secretion of the IL-8 chemokine and macrophage migration inhibitory factors (MIF) but increased also the life span and the anti-bacterial activity of neutrophils (33). Similar results were obtained with human BM-MSCs and ASCs. Indeed, MSCs increase recruitment and direct migration, increase life span and enhanced phagocytosis and respiratory burst activity of neutrophils. Effects of MSCs are dependent on soluble factors such as GM-CSF or INF-γ (34), IL-8 and MIF (33) as well as exosomes secretion (35, 36). MSCs can also display an indirect effect on neutrophil by modulating other immune cells as demonstrated by Hu et al. showing that MSCs induced IL-17 production by activated T cells stimulate neutrophil phagocytic activity (37).

Other innate immune partners that strongly interact with MSCs are monocyte and macrophage cells. Via direct and indirect effects, MSCs thus promote a pro-resolutive environment. *In vitro* studies demonstrated that MSCs trigger the polarization of macrophages from a pro-inflammatory M1 phenotype to an anti-inflammatory-like M2 phenotype (38) through the production of immunosuppressive molecules and metabolites, such as prostaglandin E2 (PGE2) (39–42) and tumor necrosis factor-stimulated gene 6 protein (TSG6) (43, 44). They also attract monocytes and macrophages through the release of chemokines such as CCL2, CCL3 and CCL12 into inflamed tissue and support wound repair (45).

This is consistent with *in vitro* data where MSCs support monocyte survival through direct interaction while increasing expression of M2 markers, phagocytic capacity and anti-inflammatory cytokines secretion *via* the production of PGE2 (46). MSCs also inhibit monocyte differentiation into dendritic cells (DC) and macrophages, decrease their phagocytosis functions and block their capacity to stimulate T cell proliferation (47). After phagocytosis of human UC-MSCs by monocytes, phagocytic monocytes acquired a predominantly anti-inflammatory phenotype characterized by increased IL-10 secretion, decreased TNF-α production and expression of M2-type receptors such as CD163 and CD206 (48).

MSCs can change the immune response locally but also change the peripheral and systemic response. In a sepsis model in mice, intravenous injected BM-MSCs interact with lung and also circulating monocytes and macrophages and reprogram them through PGE2 production. PGE2 bind to prostaglandin receptor subtype 2 (EP2) and 4 (EP4) on macrophages, producing higher amounts of IL-10. IL-10 seems prevent neutrophils from migrating into tissues limiting organ damage and reducing inflammation (2). Similarly, in an *E.coli*-induced pneumonia model, the antimicrobial effect of intravenous injected human BM-MSCs is dependent on alveolar macrophages. Indeed, the transfer of mitochondria from MSCs to macrophages, through tunneling nanotube and microvesicles, improves mitochondrial function and metabolism that enhances macrophage phagocytic capacity (49). In an experimental silicosis model, intravenous injection of MSCs-derived exosome containing miRNA or mitochondria suppress inflammation by inhibiting inflammatory monocyte recruitment in the lung. Moreover, MSCs-derived exosomes block TLR signaling in macrophages and therefore the production of inflammatory mediators linked to these pathways that are essential to the inflammatory response (50). The effect of MSCs on systemic inflammation is also true in a pathological context different from those of infection. In the phase I clinical trial ADIPOA, patients with severe knee osteoarthritis (OA) received one single intra-articular injection of autologous ASCs. Results demonstrated that after 3 months, ASCs local injection modulates the distribution of circulating monocyte subset with a significant impact on classical monocyte (51). Moreover, different studies demonstrated that intra-articular injection of MSCs decreases the pain of the patients that can be linked with a decrease of local inflammation (52, 53).

## MSCs and Immune Cells Mechanisms of Communication to Exert Immunomodulation

The constant interaction between MSCs and the innate immune system is essential to balance the inflammatory response to maintain and restore tissue homeostasis. Several channel of communication have been described.

### Paracrine Secretion

MSCs-mediated immunomodulation works through a synergy of mechanisms dependent on cell contact and soluble factors. Secretion of Interleukin 1 Receptor Antagonist (IL1-RA) (54) and PGE2 (42) can promote the polarization of macrophages toward the type 2 phenotype. Human ASCs-produced-TSG6 reduce the inflammatory response and improves DSS-induced colitis by inducing polarization towards M2 macrophages in mice (55). Similar effects were observed after MSCs treatment of inflammatory diseases such as corneal inflammation (56), acute lung injury (57) and acute peritonitis (58). TSG6 produced by MSCs also inhibits the recruitment of macrophages and inflammatory monocytes in the inflammatory environment (59). Others paracrine factors modulate also monocyte activation state. Indeed, secretion of IL-6 (60) and HGF (61) by MSCs induces the polarization of monocytes toward an alternative phenotype characterized by secretion of high level of IL-10 and low level of IL12 and TNF-α. Recent studies demonstrate that MSCs, through synergic secretion of CCL2 and CXCL12, induce M2 polarization of tissue macrophages that are necessary mediators of protective effect on colitis (62).

### Direct Cell-Cell Interaction

Education of innate immune cells by MSCs is also mediated by membrane receptor and cell-cell interactions (63). CD54 is the highest expressed adhesion molecules on MSCs and it expression is increased by inflammatory molecules (64). Several studies demonstrated that CD54 switch macrophage polarization through CD54-LFA-1 interaction. In a mouse co-culture system, Takizawa et al. demonstrated that CD54-LFA1 interaction between MSCs and macrophages promoted the proliferation of M2-macrophages under hypoxic condition (65). Interestingly, unconventional but functional interaction through CD54 between pro-inflammatory classically activates macrophages and BM-MSCs modifies MSCs functions and increases their immunosuppressive capacities (66). Moreover, CD54^high^ MSCs increase the survival of mice in a GVHD model by migrating to secondary lymph node where they suppress DCs maturation and Th1-differentiation of CD4+ T cells (67). MSCs also express receptors such as galectins that have been related to the immunosuppressive functions of MSCs. Thus, the galectin 9-expressed MSCs modulates T cell functions in particular inhibits their activation but stimulates their apoptosis (68, 69). In parallel, the direct effect of galectin 9 expressed by MSCs on the innate immune cells was not investigated. However, several studies demonstrated that galectin 9 promotes the differentiation of macrophages toward an anti-inflammatory profile (70, 71). The CD200-CD200R axis represents another specific interaction between MSCs macrophages. CD200 can bind to its receptor, CD200 receptor (CD200R), to suppress the activity of many immune cells, especially macrophages (72). CD200 expressed by human BM-MSCs inhibits the TNF-α secretion from THP-1 differentiated macrophages (73). MSCs also inhibit the differentiation of myeloid precursors through CD200-CD200R1 interaction and decrease inflammation and inflammatory cells infiltration in a corneal injury model (74). CD200R is also expressed on the microglia and recent study demonstrated that, in a model of middle cerebral artery occlusion, intravenous-injected BM-MSCs inactivate the microglia in the peri-infarct area *via* CD200-CD200R signaling pathway and reduce the volume of the infarct zone (75). MSCs can also modulate microglia activation through others receptors such as PDL-1 (76) and CXCL-4.

### Extracellular Vesicles (EV) and Exosomes

MSCs can communicate with nearby cells through secretion of exosomes, small membrane vesicles that contain bioactive molecules like proteins, lipids, mRNAs, microRNAs and mitochondria (69). These molecules can be transferred and reprogram the recipient cells (77). Several studies demonstrated that MSCs, through secretion of exosomes, modify macrophage phenotype and have beneficial effects on inflammatory diseases.

In fact, in an inflammatory bowel diseases model, infusion of MSCs-derived exosomes decreases the severity of acute but also chronic colitis by maintaining intestinal barrier integrity and reducing inflammation by targeting colon macrophages (78–81). Several papers demonstrate that the beneficial effect of MSCs-derived exosomes is mainly relied on their effect on macrophages. Indeed, Liu et al. demonstrated that metallothionin-2 containing exosomes are transferred to colon macrophages that polarize into M2 anti-inflammatory phenotype and produce IL-10, key factors of the protective effects of MSCs-derived exosomes (3, 78). Moreover, transfer of miR146 through EV-derived MSCs modify also macrophage polarization and attenuate colitis. Indeed, miR146 inhibited TNF receptor-associated factor 6 (TRAF6) and IL-1 receptor-associated kinase 1 (IRAK1) expression, down-regulated phosphorylation of NF-B p65 that decreased pro-inflammatory cytokines production by colon macrophages (80). Transfer of miR146 containing exosome from MSCs to macrophages also protects mice from sepsis by inducing M2 polarization and by decreasing inflammation (82).

Different studies also demonstrated that MSCs-derived exosomes have regenerative properties after myocardic infarction (83), acute skeletal degeneration (84) and cutaneous wound healing (85, 86) by decreasing local inflammation and by activating parenchymal cells. In lung inflammatory diseases, MSCs-derived EV protect the lung epithelial cells dependent on miR21-5p transfer, promote the polarization of alveolar macrophages from pro-inflammatory to anti-inflammatory phenotype and decrease the macrophage-driven inflammation (87, 88). The protective effects of MSCs-derived EV or MSCs alone as treatment of lung diseases and specifically acute respiratory distress syndrome (89) make them potential candidates for Covid-19 treatment (90).

Future directions already consider MSCs-EV as an alternative to MSCs cell therapy. To date, compelling evidence for safe, consistent and controlled productions is still needed in addition to proof of efficacy in preclinical studies.

### Mitochondria Transfer

Mitochondria transfer recently appeared as novel intercellular signaling pathways providing danger/rescue signals. Since tunneling nanotubes description as a possible intercellular organelle transport system (91) and the fact that such exchange can rescue aerobic respiration in cells with impaired mitochondria (92), mitochondrial transfer has been considered as an efficient way to enhance cell survival and reprogramming. MSCs are shown to release healthy mitochondria as a pro-survival factor. Conversely MSCs can capture mitochondria from damaged cells to set-up adaptive response, both mechanisms acting for cell rescue and tissue recovery (93, 94). To date intercellular transfer of mitochondria is a novel mechanism able to restitute bioenergetics in impaired cells with substantial consequences on cell fate and function opening to promising therapeutic potential. The immune and metabolic consequences of such mitochondrial exchanges will be develop later in the review.

### Efferocytosis

Clearance of apoptotic cell by macrophage and dendritic cells, i.e. efferocytosis, is reported to suppress inflammation, mediate immune tolerance and orchestrate tissue homeostasis. Interaction of macrophages with apoptotic cells leads to macrophage reprogramming and induces IL-10, TGF-β and PGE2 secretion that further blocks inflammatory mediator production whereas pro-resolutive lipid mediators are produced (such as resolvin and lipoxin) (95, 96). During acute inflammation, neutrophils massively undergo apoptosis and their uptake by macrophages reprogram them from inflammatory M1 to pro-resolutive M2 phenotype initiating the resolution phase of the inflammatory process (97). Interestingly MSCs are able to phagocyte apoptotic cells. Phagocytosis of apoptotic cells by MSCs is also reported to participate to immunosuppression through a COX2/PGE2 pathway (98). Another underlying mechanism of action in MSCs immunosuppressive activity involving apoptosis was recently revealed by Akiyama et al. MSCs secrete MCP1 to recruit and to induce T cell apoptosis *via* a FAS/FAS ligand pathway. Subsequently apoptotic T cell phagocytosis by macrophages lead to TGF-β secretion causing regulatory T cell upregulation thus resulting in immune tolerance (99).

When MSCs undergo apoptosis, they can be eliminated by macrophages. Galleu et al. demonstrated that MSCs after infusion in a mouse model of GVHD undergo apoptosis through a cytotoxic activity and still exert immunosuppressive activity. Injection of apoptotic MSCs and their phagocytosis by macrophages induce an IDO-dependent immunosuppression (48, 100). Such findings bring interesting insight in the explanation of MSCs mechanisms of action in cell therapy. These data support the hypothesis that MSCs do not necessarily have to engraft to bring therapeutic benefit. In this study, it is shown that despite their transient presence after administration, MSCs trigger efficient immunosuppression.

### MSCs Perception by the Immune System: From a Privileged to an Elusive Status

In line with this close and reciprocal interaction with immune cells and their immunomodulatory properties, MSCs have been reported to be “immunoprivileged” suggesting there could be protected against immune detection and rejection when injected in allogenic setting. To date it is considered that MSCs are rather “immunoevasive” meaning weakly immunogenic (101). It means that the inflammatory tissue context influences the balance between immunosuppressive activity and MHC Class II expression by MSCs that drive the immune reactivity leading in either tolerance or rejection after allo-immunization. Nevertheless, and as described above a prolonged persistence of MSCs is not necessarily required for a sustained therapeutic effect. In some pathological situation, a transient presence could be sufficient to guide tissue cell behavior for a long lasting therapeutic benefit (102, 103). It thus appears that further data are required to understand how host immune response to allogenic MSCs occur and how it may affect MSCs therapeutic efficacy in acute syndromes as well as chronic diseases. MHC controls, MSCs immunogenicity through appropriate immuno-monitoring should bring answers.

## MSCs as a Full Non-Hematopoietic Immune Cells Component?

It is admitted that immune functions are however not restricted to hematopoietic cells and also include stromal or structural cells, and MSCs as such, that are able to express a large range of immune regulators and cytokines. It is noticeable that MSCs may also trigger some immune functions by themselves, such as mechanisms against pathogen defense that can, to some extent, be compared to professional phagocytes.

MSCs are currently considered as new possible treatment for infectious diseases link to their antimicrobial activity (104, 105). Indeed, INF-γ and TLR activated MSCs can improve the antimicrobial activity of certain antibiotics (106) and secrete antimicrobial peptides such as cathelicidin LL-37 (105, 107), human β-defensin-2 (hBD-2) (108), hepcidin (109), and lipocalin-2 (Lcn2) (110). Different studies demonstrated that untreated MSCs also have an antibacterial activity. Monsarrat et al. showed that ASCs could disturb bacterial division, induce bacterial membrane permeabilization through ROS secretion and phagocyte Gram-positive and Gram-negative bacteria. Moreover, in a mouse model of pathogen-induced periodontitis, engraftment of human ASCs decreases the bacterial load (111). Unstimulated conditioned medium of human BM-MSCs shows an antimicrobial activity as well as an inhibitory effect on biofilm formation of *V. cholerea* (112).

However, the major antibacterial effect of MSCs remains linked to the modulation of the immune system. First, MSCs can produce chemokines such as MIP1α (macrophage inflammatory protein 1-alpha) and CXCL2, which attracts pro-inflammatory M1 macrophages that have antimicrobial activities (45, 113). Additionally, MSCs can increase macrophage phagocytosis capacity *in vitro* and *in vivo* of mouse alveolar macrophages and human monocyte-derived macrophages linked to the transfer of mitochondria. In a mouse model of *E. coli*-induced pneumonia, the antimicrobial effect of intravenous-injected MSCs is dependent on alveolar macrophages and on the nanotube and microvesicles-mediated mitochondrial transfer from MSCs to alveolar macrophages (49). Transfer of mRNA through microvesicle from MSCs to macrophages can also modulate antibacterial immune response. Indeed, in an infectious model of acute lung injury, intravenous administration MSCs microvesicles 4 hours after the injury reduces the influx of white blood cells and neutrophils and decreases the total protein concentration in the broncho-alveolar lavage fluid at 24h (114).

MSCs can also be efficient as carrier to deliver antibiotics. BM-MSCs are capable to uptake ciprofloxacin and then release active antibiotic up to 24 hours to inhibit bacterial proliferation (115). Several studies demonstrated that MSCs have higher engraftment capacity within sites of inflammation and bacterial infection therefore antibiotic-load MSCs can represent a puissant tool to deliver antibiotics into infected deep tissues. However, to firmly rely on such potential additional *in vivo* experiments are required.

To move forward, the next provocative question would be can MSCs be considered as macrophage-like cells? This striking question takes its roots in seminal paper describing that preadipocytes/ASCs share numerous features with macrophages such as inflammatory cytokine release and phagocytosis of apoptotic bodies (116) that was also confirmed in human (117). At this time, ASCs were named preadipocytes because MSCs were restricted to bone marrow and were not yet the object of many attentions for their biological roles and the perspectives they may open in regenerative medicine. The close similarity between adipose progenitors and macrophages has been emphasized later (118) and described as related to stemness of cells (119). It is noteworthy that the putative link between adipose progenitors and innate cells is consistent for a phylogenetic point of view with the well-known role of fat body cells in the defense system of insects (120). This striking analogy is also highlighted by the fact that adipose progenitors proliferate upon inflammatory cytokines stimulation (121) and furthermore, can be infected and represent a reservoir for different infectious agents among which HIV, trypanosoma and mycobacterium tuberculosis (122–124). This privileged link with infectious agents could be due to the emerging role of intracellular lipid micro-droplets as assembly platforms for pathogens (125, 126). Whereas this literature is essentially based on investigations on adipose tissue and could thus be considered specific to ASCs for their commitment towards adipocyte lineage, recent publications show that such conclusions can be extended to MSCs (127). Recent multi-omics comprehensive profiling brought strong arguments to consider such stromal cells as key players in the immune system (128). An extensive characterization in twelve organs in mice is proposing that structural cells, i.e. epithelial, endothelia and fibroblast cells largely regulate immune genes transcription and epigenetic regulation upon infection and may thus be considered as an integral part of the immune system, together with hematopoietic cells (128). Although the term of MSCs is not used the close relationship between MSCs and fibroblast including in cancer context (129), this work largely claims a direct role of MSCs in immunity as active immune agents and not only as a partners of classic hematopoietic immune cells.

Finally, the analogy of MSCs with macrophages extends to their phenotypic polarization as inflammatory cytokines such as INF-γ, TNF-α or TLR3 stimulation primes MSCs into an anti-inflammatory and immunosuppressive MSC-2 phenotype (secreting IDO, NO, PGE2, TGF-β) whereas TLR4 activation rather induces a pro-inflammatory MSC-1 phenotype (producing MIP-1α, MIP-1β, RANTES, CXCL9, CXCL10) (130, 131).

## Metabolic coupling of MSCs with Immune Cells and Functions

Cellular metabolism supports all tissue functions ensuring on demand energy supply required for cell activity. Additionally, nutrient-derived metabolites are important mediators of cellular metabolic fluxes and signaling. Both immune and metabolic functions are regulated by common pathways that act in concert when sensing tissue threats to provide a coherent adaptive response converging organism resources towards an appropriate energy production for vital functions such as tissue defense and regeneration. Inflammatory signaling factors (i.e. cytokines, lipid mediators, amino acid-derived metabolites, pathogen-mediated signals, tissue danger signals) and hypoxia can orientate metabolic pathways in various cells other than immune cells, adipocytes, hepatocytes, neurons and MSCs for example.

### Intracellular Metabolism Drives Fate and Functions in MSCs and Immune Cells

Immunometabolism classically refers to immune cells metabolism that governs their fate and function. It is greatly documented how cellular metabolic fluxes and metabolite detection dictate the immune response developed by macrophages, dendritic cells, neutrophils and lymphocytes (132–134). It is now admitted that macrophages polarization is connected to metabolic pathways with an activation of glycolytic pathways in inflammatory M1 macrophages whereas M2 macrophages use fatty acid oxidation and mitochondrial oxidative phosphorylation system (OXPHOS) reactions to generate ATP (135, 136). It is reported that metabolic reprogramming can operate in immune cells as a result of tissue factor detection and oxygen availability. Oxygen deprivation, similarly to LPS exposure, induce an increase in HIF-1α associated with a glycolytic pathway activation leading to a M1 polarization. In M1 macrophage where glycolytic pathway dominates, mitochondrial ROS production also increases (via succinate accumulation and oxidation) thus sustaining the inflammatory status (137). Conversely an increase in PHD2 activity, as observed under physioxic condition, or an increase in HIF-2α are associated with a switch towards a M2 phenotype and an elevated fatty acid oxidation and mitochondrial respiratory chain activity (138). Such striking impact of metabolism is not only true for innate but also for adaptive immune cells and can largely be extended to MSCs. Metabolic fitness of MSCs in response to environmental signals regulates their immunomodulatory properties suggesting that immunometabolism also applies to MSCs.

### Metabolism at the Heart of MSCs and Immune Cells Crosstalk

Evidences of direct metabolic coupling between MSCs and immune cells are poorly documented. It is just demonstrated that human MSCs induce the acquisition of M2 macrophage phenotype through a lactate-mediated metabolic reprogramming (139). Reciprocal metabolic reprogramming is more described between stromal and cancer cells. It is largely described how cancer cells hijack stromal cells activity to develop metabolic adaptive strategies based on oxidative stress handling, to favor tumor promotion (140, 141).

Indirect coupling evidences are more obvious in the literature. MSCs are sensitive to micro-environment changes appearing with tissue dysfunction and can operate metabolic switch and functional adaptations in their biology that in turn will impact the immune response and inflammatory status. Modulation of the redox environment and oxidative stress resulting from free radical accumulation (reactive oxygen species, ROS and reactive nitrogen species, RNS), pH, oxygen tension and nutrient gradient perturbations are associated with cell injury, organ failure and the aging process (142, 143). MSCs are quite resistant to oxidative stress and react by developing strong enzymatic (superoxide dismutases, glutathion peroxidase, catalase, sirtuins, heme oxidase-1) (144–147) and non-enzymatic (reduced glutathione, HIF-1α, Heat Shock Proteins, nuclear factors) (148–150) scavenging, anti-oxidant and cytoprotective defenses. These activities that modulate redox status are concomitant with the immunomodulatory effect of MSCs; some being required as demonstrated with depletion/deletion, overexpression approaches (145, 151, 152) or pre-conditioning strategies (86, 153–157), some being induced downstream of immune pathways (158).

Mitochondria thus appears to play a pivotal role in immune cell metabolic reprogramming ensuring, as a signaling hub, the adequate crosstalk between immunity control and cellular energy providing (137, 159–162). To that end, mitochondria transfer is under investigation. Mitochondrial exchange may occur through tunneling nanotubes, microvesicle capitation, cell junction, cell fusion or direct uptake (93, 94). MSCs can donate healthy mitochondria to damaged cells rescuing aerobic respiration in cells with mitochondrial genetic defect (92) and reprogramming and restoring bioenergetics needs of injured cells in various disorders such as brain stroke, ischemic heart, lung, kidney and degenerative diseases (163–166). Mitochondrial translocation from MSCs to immune cells can trigger immunomodulatory mechanisms. Mitochondria transfer from MSCs to macrophages enhance phagocytosis and anti-inflammatory phenotype (49, 167). The uptake of MSCs mitochondria by T helper 17 cells promote their acquisition of anti-inflammatory phenotype and their metabolic reprogramming towards OXPHOS reactions (168). According to such findings mitochondria transfer from MSCs mediates tissue repair and regeneration and take part in their therapeutic potential. It is noteworthy that bidirectional mitochondrial exchange can operate. When damaged or stressed, cells release and convey mitochondria to MSCs and it is shown to activate anti-apoptotic function and mitochondrial biogenesis in MSCs (169). MSCs exposure to mitochondria from damaged cells that confer cytoprotective and pro-regenerative properties may be a way to precondition MSCs for an enhanced and targeted therapeutic efficacy. Nevertheless, the mechanisms of mitochondrial transfer through GAP junctions, nanotubes, uptake, cell fusion or micro-vesicles, as well as the frequency, the efficacy and the donor cells have to be further explored to give rise to therapeutic approaches using mitochondrial transplantation (93). In addition, mitochondrial transfer has to be manipulated with caution due to dual effects of metabolic switch that may promote repair but cancer as well (170).

It was recently reported that liver tumor cell growth was associated with mitochondrial fusion causing an increase in oxygen consumption and ATP production (171). The possibility that metabolic status can be inferred from mitochondrial morphology opens to numerous perspectives in MSCs therapy. Indeed, mitochondria functions are intimately linked to their morphology, where fission causing mitochondria fragmentation is associated with metabolic dysfunction whereas fusion generating a functional network is acting to preserve and protect cell integrity. In MSCs, mitochondrial dynamics is already known to be involved in metabolic changes and senescence favoring tissue recovery and regeneration (172, 173). Additional studies are still necessary to confirm a causative relationship between MSCs activity and mitochondria morphological changes.

Other metabolic pathways in MSCs immunomodulatory activity relies on enzymes degrading amino acids and producing metabolites that may boost or dampen immune responses. Tryptophan is an essential amino acid catabolized through the kynurenine pathway by the rate-limited enzyme indoleamine 2,3-dioxygenase (IDO). In MSCs the activation of IDO in response to inflammatory factors is triggering immunosuppression as a result of tryptophan depletion causing apoptosis of T cells, inhibition of T cells and NK cells proliferation, inhibition of DCs maturation, and induction of Treg immune suppressor cells (174). On the other hand, tryptophan metabolites from the tryptophan hydroxylase (TPH) pathway (5-methoxytryptophan, melatonin) protect MSCs against replicative and oxidative stress-induced senescence (175). The amino acid arginine is catalyzed by enzymes highly regulated by the inflammatory setting leading to opposite immunological consequences in myeloid cells. Indeed, in inflammatory M1 macrophages arginine is catabolized by nitric oxide synthases (NOS) into NO and citrulline. While in wound healing M2 macrophages, arginine is rather metabolized by arginases generating ornithine, urea, polyamine and proline (176). Whether such decisional amino acid-dependent metabolic switch exists in MSCs is not known and has to be explored.

**Figure 2:**
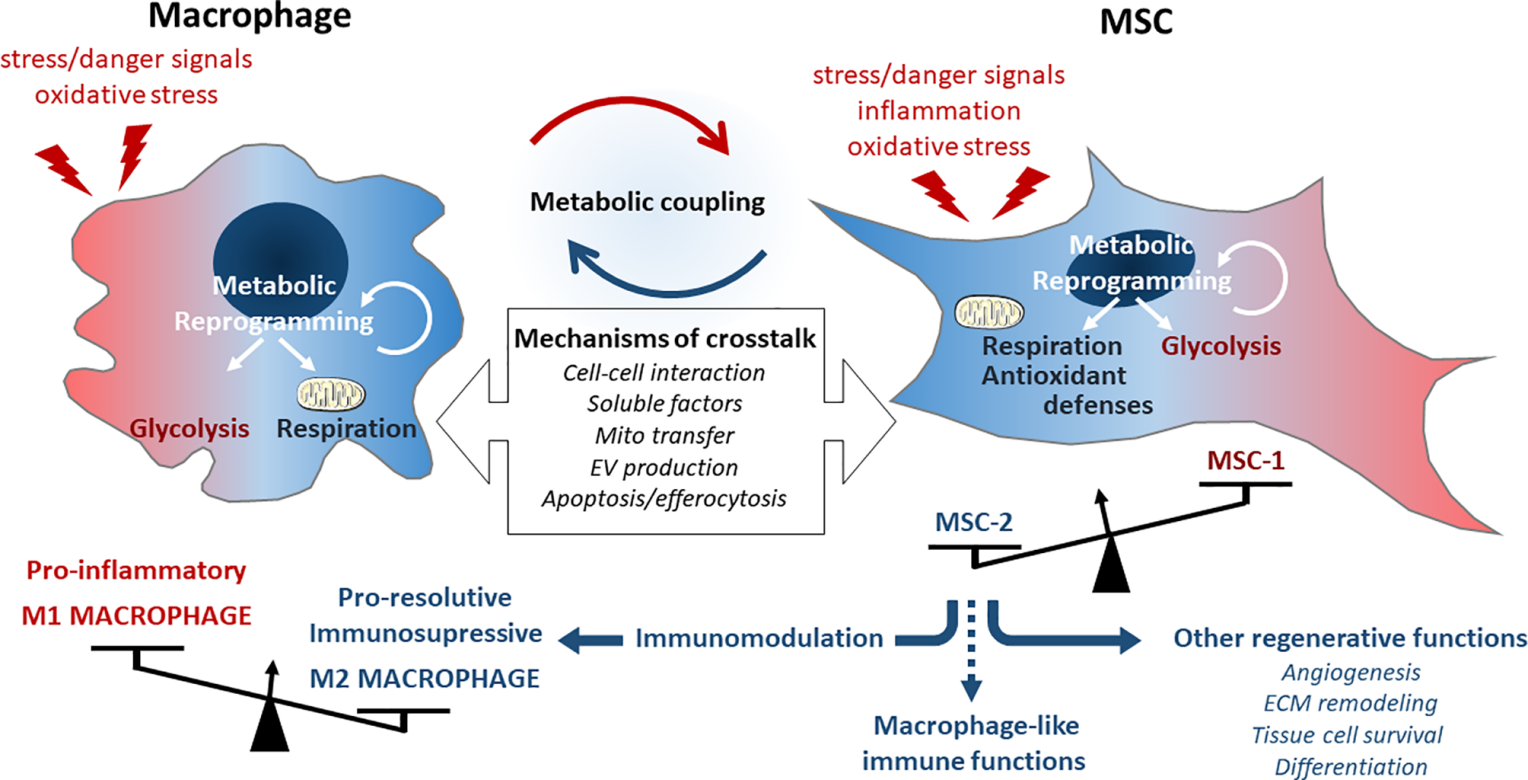
Description of the metabolic immuno-mesenchymal axis. Macrophages can be activated to polarize into M1 phenotype driving the inflammatory response upon cell damage or tissue failure. This phenotype is associated with a glycolytic metabolism and the production of multiple inflammatory and oxidative signals detected by MSCs through different way of communication. MSCs react by functional adaptations of their metabolic and immune functions but will basically induce macrophage switch into a M2 phenotype associated with their metabolic reprograming and develop immunosuppressive and regenerative activities.

Finally, mesenchymal and immune cells that both act as sensor and actor of tissue integrity, are tightly connected entities where their respective metabolic and functional activities result and influence each other. They both constantly integrate environmental signals and communicate for a concerted and efficient metabolic reset to properly fuel functional adaptations of the immune response (**Figure 2**).

Ultimately, immunometabolism has to be investigated at the whole organism level considering its generalized high impact effect in homeostasis as well as pathogenesis and aged-related chronic diseases (132). Systemic immunometabolism exploration and control, possibly through targeting MSCs activity, represent the current challenge to develop integrated and holistic approaches for regenerative medicine strategies.

## Challenges and Opportunities for Future therapeutic Applications

Many unsolved questions remain to overcome present limitations to reach sustain and significant efficacy that is not yet achieve in late-phase clinical trials. The translation of MSCs into clinic is more difficult than expected. Indeed, the cell therapy outcome is not solely depending on ATMP properties but also conditioned by the route of delivery. This major issue is currently questioned and must be considered as an adaptable option to fit with the multiple possible applications (177). Intravenous infusion of MSCs is the most common way due to the convenience and limited risk, and remains appropriated when systemic effects are expected. However, several groups demonstrated that intravenous-injected MSCs are trapped in the lung and persist less than 3 days in the tissue (178, 179) whereas local application or injection in the targeted tissue seems to increase engraftment. Intramuscular-delivered human MSCs in mouse models survived for up to 5 months after injection (180). In a comparative study, Giri et al. observed that extravascular injection of MSCs improves colitis while the intravenous injection at maximal dose does not (181) suggesting that cell delivery is a critical issue for therapeutic efficacy. Satisfactory solutions may arise from recent developments that associate cells with smart materials, bio-printed scaffolds or organoids allowing 3D structuration opening to alternative approaches for tissue filling, replacement and topic applications, with the advantage of including the biomechanical component required for guiding an optimal tissue reconstruction. Whether MSCs need to integrate the targeted tissue, are intended to be progressively replaced native tissue cells or have to rapidly undergo apoptosis for optimal therapeutic benefit is not resolved. Such apparent contradictions have to be addressed and certainly reveals the existence of multiple pathology-dependent mechanisms of action that may ultimately lead to the same outcome.

Besides, in order to succeed in prompting MSCs therapy into suitable clinical practices, the current major challenges reside in (i) MSCs manufacturing towards standardized and optimized culture processes, (ii) improving the MSCs characterization including multiscale and multiparametric analyses, (iii) developing dynamic potency tests to qualify MSCs batches that bring guaranties of their therapeutic potential, (iv) identifying from completed trials MSCs parameters correlated with clinical benefit in order to propose predictive markers for a given physio-pathologic background. The study of MSCs biology over the last decade underlines that beyond their intrinsic potential (availability, tissue specificity (182), individual features from global multi-omic and phenotyping signatures) we have to estimate, for clinical purpose, their wide range of responses when facing a challenge. Their tissue function and therapeutic benefit indeed reside in this unique ability to map environmental cues, orchestrate local reactions with possible systemic consequences such as immunometabolism reprogramming.

Bio-production and bio-banking processes for clinical purpose impose environmental conditions that can change or alter biological properties of MSCs and therefore have to be carefully establish to not compromise their therapeutic potential. Most of the clinical trials used cryopreserved MSCs that were defrosted and immediately infused to patients. However, numerous studies demonstrated that MSCs immediately post-thaw have less immunomodulatory capacity *in vitro* (183, 184). They are more susceptible to T cell-mediated lysis as well as instant blood mediated inflammatory reaction (178, 185) that can explain the low persistence of cryopreserved-MSCs after injection. Interestingly, a culture step prior the implantation rescues post-thaw deleterious effects, reverses cryoinjurious effects (186, 187) and increases the engraftment of the cells in tissue (180).

Confirming the importance of such considerations, it can be noticed that the only MSCs-based ATMP approved by the European Medicines Agency is Alofisel^®^ to treat Crohn disease. The indication is the use allogenic ASCs cultured prior the implantation and locally administrated into the anal fistula (188, 189). Cell therapy procedure is thus validated as a combination of a pharmaceutical cell product, a production process and a mode of delivery all driven by the specificity of the pathological context to be treated.

MSCs qualification is the next step to improve. Dynamic tests exploring their plasticity (stimulate/inhibit a function) (190, 191), flexibility/stiffness (amplitude in resistance and response to stress) and decisional metabolic switches should be more predictive regarding to therapeutic expectations. Such functional characterization may also influence the bio-production process for MSCs and methods to acquire, preserve and/or enhance the expected potency after administration. Optimized cell production can be reached through MSCs preconditioning or licensing (transient hypoxia, cytokines priming, nutrients stress before administration) or at least may define the most appropriate therapeutic window for MSCs therapy (192–197).All these datasets integrating and combining multi-parametric individual characteristics with therapeutic potential assessed *in vitro* will allow to correlate single-cell signatures with collective response to a challenge at the cell population level to define bioengineered MSCs product fitness. Computational analysis including principal component analysis, correlation, clustering analyses together with Machine Learning methods should help to resolve the question of cell heterogeneity and functional potency to propose customized productions.

A next critical issue resides in being able to select the most permissive cell product for personalized therapeutics benefit according to patient characteristics. Indeed, extensive analyses of MSCs properties *in vitro* is not sufficient to predict their efficacy in patient. Retrospectives analyses of clinical outcomes and recovery in cohorts of patients receiving allogenic MSCs should allow to identify specific markers or signature associated with MSCs treatment benefit. Such approaches should help to address the question of responder and non-responder identification. Based on clinical trial data and MSCs testing *in vitro*, Artificial Intelligence and Deep Learning models could be trained to predict therapeutic MSCs efficacy in a given pathologic background.

### Aging Effects on MSCs Properties

With aging the complex and dynamic networks involving native MSCs activity may gradually deteriorate due to alteration accumulation in MSCs and/or modification of their surrounding environment. Declines that progressively operate may at some point exceed MSCs resilience capacity and ultimately compromise tissue homeostasis and functional integrity (198, 199). With aging changes in individual features of MSCs are observed such as a decrease in number, in differentiation potential, in proliferation rate, in redox control, some mitochondrial dysfunctions and modifications of their secretory repertoire associated with a senescent phenotype (200–202). MSCs age-related dysfunction may alter MSCs/immune cells dialogue and lead to a loss in their immunomodulatory activity and therefore negatively impact therapeutic outcomes in cell therapy (203–206). The question of MSCs senescence and resilience is not sufficiently addressed until now and should be implemented to the required safety control performed to release suitable MSCs for clinical use. Another critical point would be to delineate the putative involvement of MSCs as a niche component in the reported progressive deviation of the hematopoiesis towards myeloid versus lymphoid lineage presaged as causative in the emergence of inflammaging (207). To some extend MSCs aging and associated functional consequences have to assess independently of the age of the donor for therapeutic use.

### Opening to Allogenic MSCs Therapy for Metabolic and Inflammatory Age-Related Diseases

The risk for developing a wide range of chronic disorders rises with aging and represent a growing public health issue with the increased lifespan of the population. The geroscience approach aims to prevent, delay and possibly reverse the occurrence or the severity of age-associated chronic pathologies by acting on key molecular, cellular and systemic aging processes. Allogenic MSCs from healthy donors thus appear as a promising candidate due to their pleiotropic effects to offer new options to treat age-related disorders associated with chronic inflammation. MSCs therapy trials in the elderly already showed benefits in osteoarthritis (208, 209), periodontitis (210), type 2 diabetes (211) or neurodegenerative diseases (212, 213) for example. Recently allogenic MSCs therapy was tested in aging frailty showing promising results displayed by a decrease in inflammatory status and an improvement in physical performance over a 6 months follow-up (214). Ongoing trials outcomes are however required to confirm those preliminary achievements.

## Conclusion

Supported by recent single cell RNA sequencing in mouse, resident immune and stromal cells organize into a cross-tissue cellular network. Together with endothelial and dendritic cells they represent the recurrent components contributing and defining each tissue micro-environment, highlighting their crucial role in local response that can extend to the systemic level (215). Stromal cells and resident immune cells belong to the connective tissue present in all organs allowing them to display a specific and adapted tissue-dependent activity as well as to integrate and modulate systemic response at the organism scale through an interconnected and dynamic network. Metabolic reprogramming is a way to shuttle signals and factors in this privileged bidirectional dialogue.

To conclude, where the reductionist approach consisting in managing each disease independently have proved to be ineffective in the treatment of age-related pathologies, allogenic MSCs therapy can be proposed as an alternative solution for patients elected through reliable criteria. Inflammation and metabolism can be considered as biological rheostat able to reset the entire system and MSCs as a determinant regulator of both functions towards homeostasis restoration. Based on relevant selection criteria and optimized production process, allogenic MSCs position as multimodal tools able to take in charge biological functions interfacing with all organs. Allogenic MSCs therapy by targeting inflammaging and metabolism-aging thus appears in line with geroscience perspectives (216) and is a major challenge to address for the upcoming regenerative and rejuvenative medicine strategies.

## Author Contributions

VB and AV conceived the main conceptual ideas and structure of the review. VB, AV and LC wrote the manuscript, analyzed and sorted the literature, revised and provided critical feedback on the manuscript. VB provided funding. All authors contributed to the article and approved the submitted version.

## Conflict of Interest

The authors declare that the research was conducted in the absence of any commercial or financial relationships that could be construed as a potential conflict of interest.
